# The Relationship of Nurse and Physician Staffing in Intensive Care Units with Patient Outcomes in Postoperative Patients on Ventilators: An Analysis Using Korean National Health Insurance Data

**DOI:** 10.3390/healthcare11081124

**Published:** 2023-04-13

**Authors:** Yunmi Kim, Seon-Ha Kim

**Affiliations:** 1 Department of Nursing, Eulji University, Seongnam 13135, Republic of Korea; 2 Department of Nursing, Dankook University, Cheonan 31116, Republic of Korea

**Keywords:** hospital mortality, intensive care units, personnel staffing and scheduling, pneumonia, postoperative period

## Abstract

This study examined the associations of nurse and physician staffing in intensive care units (ICUs) with hospital-acquired pneumonia (HAP) incidence and in-hospital mortality in postoperative patients on ventilators. National Health Insurance claims data and death statistics were used to investigate the nurse staffing level and the presence or absence of a dedicated resident and specialist in each ICU. The participants were patients aged 20–85 who underwent any of 13 surgical procedures and were placed on a ventilator in the ICU after the procedure. Of 11,693 patients, 307 (2.6%) experienced HAP and 1280 (10.9%) died during hospitalization. Compared to hospitals with lower nurse-to-patient ratios, patients in hospitals with higher ratios had statistically significantly higher risks of HAP and in-hospital mortality. The presence of a dedicated resident in the ICU did not statistically significantly affect HAP incidence or in-hospital mortality. The presence of an ICU specialist was statistically significantly associated with in-hospital mortality but not HAP incidence. Our findings suggest that a higher level of nursing staff in the ICU is inversely associated with HAP incidence. The legal standards for nurse staffing in the ICU should be strengthened in order to improve the quality of care and patient safety.

## 1. Introduction

Intensive care units (ICUs) provide intensive management for patients at high risk of death due to severe disease. The medical and nursing services provided in ICUs have a major influence on the quality of individual hospitals and the healthcare system as a whole. The ICU mortality rate in developed countries ranges from around 10% to 30% [1], and the in-hospital mortality rate in ICU patients in South Korea (hereafter Korea) has been reported to vary from 12.8% to 14.5% from 2009 to 2014 [2]. An ICU must have appropriate personnel and systems to closely monitor critically ill patients and engage in clinical interventions with the available resources within a short period of time [3]. Therefore, ICUs require much higher staffing of physicians and nurses than general inpatient wards, and the operating costs of ICUs are high due to the use of expensive medical equipment, tests, medicines, and treatments. In Korea, staff salaries are the costliest items in most medical budgets and the level of reimbursement for critical care management is generally low. For this reason, hospital managers are reluctant to invest in ICU personnel [4]. A report on ICUs in the United Kingdom found a threefold or higher difference in nurse staffing and nursing costs at medical institutions depending on the size of the ICU [5]. Although 25% of medical expenses in Korea are devoted to the treatment of critically ill patients, hospitals are reluctant to invest in ICUs due to their high cost and low revenue [6].

Some previous studies have reported conflicting findings regarding the relationship between the level of nurse staffing in the ICU and in-hospital mortality in ICU patients [7,8], while others have reported that the level of nurse and physician staffing in ICUs had a statistically significant association with mortality in ICU patients [5,9,10]. In a recent systematic review of the relationship between the variation in registered nurse staffing in the adult ICU and patient outcomes, studies with strong internal validity reported significant associations between lower levels of critical care nurse staffing and increased odds of both patient mortality (1.24–3.50 times greater) and healthcare-associated infections (3.28–3.60 times greater) [11]. It has been reported that the level of nurse and physician staffing is related to ventilation-associated pneumonia and mortality [12]. Regarding ICU physician staffing in Korea, the in-hospital mortality rates were reported to be 16% and 20%, respectively, in patients treated at hospitals with and without a dedicated ICU specialist in the ICU [13].

In Korea, 35% of ICU patients use a ventilator during their stay in the ICU [14]. Patients on ventilators are not only vulnerable to systemic conditions but also have a high risk of developing ventilator-associated pneumonia (VAP), which increases the risk of mortality [15,16]. A meta-analysis found that VAP was associated with a threefold increase in mortality compared to patients who did not develop VAP [17]. In a meta-analysis of 9 case-control studies, the odds ratio (OR) for ICU mortality in patients with VAP compared to those without VAP was 1.94 [18]. The in-hospital mortality rate of ICU patients on ventilators in Korea has been reported to be 48%, dramatically exceeding the rate (11%) of those not on ventilators [13].

The mean daytime nurse-to-patient ratio, which was obtained by dividing the number of patients during the day shift by the number of on-duty nurses, in Korea’s ICUs in 2014 was 1:2.7 [19]. In developed countries, the regulations or recommendations for nurse-to-patient ratios in ICUs vary by country and among specific regions within a single country, but most recommend a level of 1:1 to 1:2 or less. [20,21,22]. The Ministry of Health and Welfare in Korea has introduced a policy of differentially paying hospitalization fees according to the level of nurse staffing to solve the problem of the low level of nursing at medical institutions. In 2008, 9 tiers (grade 1 to grade 9) of reimbursement for ICU nursing management fees were introduced according to the number of nurses in adult ICUs. In 2015, the Ministry of Health and Welfare changed the system for tertiary hospitals to 5 tiers of reimbursement (grade 1 to grade 5, standard: grade 4). The Korean nurse staffing grades in the ICU are determined using the nurse-to-bed ratio, where a lower grade means a higher level of nurse staffing. The details of the grades of nurse staffing are described in the Methods section. The magnitude of the additions and subtractions of nursing management fees according to the grade was increased. For example, if a patient is admitted to the ICU with grade 4 nurse staffing at a tertiary hospital, a nursing management fee of 154.5 USD (1 USD = 1200 KRW) per patient per day is paid. The daily ICU fees for grades 3, 2, and 1 increase by 15%, 30%, and 45%, respectively. Those of grade 5 decrease by 15% compared with grade 4 (Table 1) [23]. In addition, the management fees for dedicated residents and specialists in critical care medicine (hereinafter specialists) in the ICU were introduced in 2008 and 2015, respectively. However, the differences in ICU management charges are not nearly sufficient to entice hospital administrators to invest in better patient care by employing more nurses per unit [6]. Although many studies have examined the relationship between ICU staffing levels and patient outcomes, those studies have been subject to limitations in terms of mixing various types of subjects and hospitals. Few studies have investigated the effects of nursing and medical personnel levels on patient outcomes in postoperative patients on ventilators.

Therefore, it is necessary to identify the differences in the patient outcomes according to the level of the nurse and physician staffing in ICUs using health insurance data. The aim of this study was to investigate the association of the staffing level with the outcomes of patients on ventilators, who are highly dependent on ICU nurses and dedicated physicians. This study examined the distribution of nurse staffing levels in the ICUs of tertiary hospitals, as well as the proportion of ICUs with a dedicated resident or specialist and analyzed the associations of these parameters with hospital-acquired pneumonia (HAP) and in-hospital mortality during hospital admission in patients on ventilators after surgery.

## 2. Materials and Methods

### 2.1. Design and Data

This was a retrospective study of existing data. The analysis was conducted using National Health Insurance administrative data and death statistics from 2015 to 2016, which made it possible to control for covariates, including patient and hospital characteristics.

### 2.2. Study Participants

The inclusion criteria were (1) people aged 20–85 years who received interventions corresponding to 12 surgical procedures and percutaneous angioplasty at 43 tertiary hospitals nationwide from 1 January 2015 to 31 December 2016 who (2) were placed on a ventilator in the ICU after surgery. The age range of the participants was determined by referring to a previous study [24]. Because all tertiary hospitals in Korea are accredited institutions, the data from tertiary hospitals have the advantage of high validity and similar patient characteristics.

Postoperative patients were selected for the study because the claims data did not contain information on requests to discontinue life-sustaining treatment. Therefore, if the analysis had encompassed all ICU patients, it would have been impossible to distinguish whether death was due to discontinuation of life-sustaining treatment or failure of resuscitation. The participants of this study included patients who underwent 12 surgical procedures known to have a high in-hospital mortality rate while undergoing surgery under general anesthesia, as well as patients who underwent percutaneous angioplasty, which has a high incidence of cardiac arrest. Patients placed under general anesthesia are unlikely to have pneumonia at the time of surgery. The target institutions were limited to 43 tertiary hospitals to ensure homogeneity of the institutions’ patient severity and medical environment.

**Figure 1:**
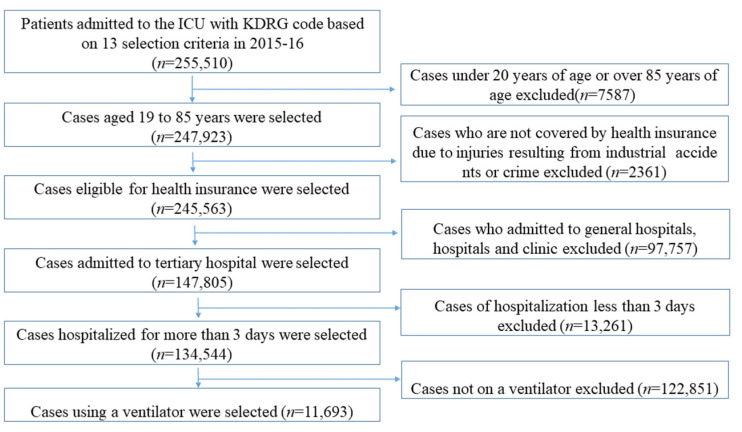
Data extraction flow.

Data on surgical groups were extracted using the following Korean Diagnosis-Related Group codes: B01*, major craniotomy; B02*, other type of craniotomy; B03*, craniotomy for trauma treatment; F02*, cardiac valve surgery using cardiac catheterization; F03*, cardiac valve surgery without cardiac catheterization; F04*, coronary artery bypass graft; F06*, major vascular repair surgery; F07*, percutaneous angioplasty; G01*, esophageal surgery; G02*, rectal surgery; G03*, major small and large intestine surgery; G04*, gastric and duodenal surgery; H01*, pancreas and liver surgery. The use of a ventilator was analyzed using the frequency of claiming the treatment codes (M5850, M5857, M5858, M5860) and categorized as <1 day, 1 to 3 days, and 4 days and more. Hospital-associated infection refers to an infection that occurred 48 h after hospitalization, so those who were hospitalized in an ICU for less than 2 days were excluded. In total, 11,693 participants were finally analyzed. The data extraction flow is presented in Figure 1.

### 2.3. Measurement

#### 2.3.1. Hospital Characteristics

The following hospital-related factors were investigated: the nurse staffing level, the presence or absence of dedicated residents and specialists in the ICU, hospital ownership, the location of the hospital, and the number of beds in the adult ICU.

Table 1 summarizes the nurse staffing levels according to ICU nursing staffing grade in tertiary hospitals. In this study, the nursing staff levels were classified into grade 1, grade 2, and grade 3 or higher. Higher grades indicate lower nurse staffing levels. A detailed explanation of Table 1 is as follows. In accordance with the standards set by the Ministry of Health and Welfare, Korean nurse staffing grades in ICU are determined quarterly using the nurse-to-bed ratio. This ratio is calculated by dividing the average number of operating beds in the previous quarter by the average number of full-time equivalent registered nurses in the ICU in the previous quarter. In the claims data, the nurse staffing level in the ICU of each institution is provided. The nurse staffing level in the ICU of a tertiary hospital is classified into grade 1 (less than 0.5 beds per ICU nurse), grade 2 (0.5 or more and less than 0.63 beds per ICU nurse), grade 3 (0.63 or more and less than 0.77 beds per ICU nurse), grade 4 (0.77 or more and less than 0.88 beds per ICU nurse), and grade 5 (0.88 or more beds per ICU nurse). Nurse-to-patient ratio is converted using nurse-to-bed ratio by applying the 94.4% bed occupancy rate of ICUs of the tertiary hospitals. The number of patients per shift nurse is multiplied by 3 times the nurse-to-patient ratio (3 shifts) and multiplied by 365 days divided by the average number of working days per year (365/230). That is, 4.8 times nurse to patient ratio is applied [25]. The severity of patients in the ICU is not reflected when calculating the number of nursing staff. There are no special qualifications or skills required for ICU nurses in Korea.

The presence of an ICU-dedicated physician (a physician who works only in the ICU and cares exclusively for ICU patients) was analyzed for general residents (claim code AJ001) and ICU specialists (claim code AJ003).

Hospital ownership was divided into (1) public, (2) educational foundations, and (3) medical corporations. The location was divided into Seoul (the capital of South Korea), metropolitan cities, and other regions. The number of beds in the adult ICU was used as a surrogate indicator in order to reflect the volume effect of hospitals, although there are limitations that do not reflect the bed occupancy rate. Data on hospital characteristics were obtained from the second quarter of 2016, the midpoint of study period. The data for the second quarter of 2016 reflect the level of nurse staffing in the first quarter of 2016.

#### 2.3.2. Patient Characteristics

Patient characteristics included age, sex, type of operation (Korean Diagnosis-Related Group codes), comorbid diseases, admission route, history of respiratory disease, and the duration of ventilator application.

For comorbid diseases, the Charlson comorbidity index was calculated using the primary and secondary diagnosis codes [26]. The Charlson comorbidity index is widely used to measure the burden of comorbidities in medical research. It assigns a score based on the presence or absence of 17 comorbidities, including congestive heart failure, diabetes, and cancer [26]. The diagnosis was coded based on the International Classification of Diseases 10th revision. The Charlson comorbidity index was classified into 0 points, 1–5 points, and 6 points or more. A higher score means more comorbidities.

A history of respiratory disease was defined based on the presence of the diagnostic code for a respiratory disease (J00–J99) within 1 month before hospitalization. HAP was defined as a diagnostic code of J13*, J14*, J15*, J16.8, J18*, J69.0, or J69.8 during hospitalization. In-hospital mortality was defined as occurring when the patient’s discharge date was the same as the death date from the National Statistical Office.

### 2.4. Data Analysis

Univariate analyses using the Chi-square test were employed to explore HAP and in-hospital mortality according to the characteristics of patients and hospitals. Logistic regression with generalized estimating equations was used to evaluate the relationship between the dependent variable, HAP, in-hospital mortality, and the level of nurse staffing and physician staffing, adjusting for the covariates described previously. Generalized estimating equations are commonly used in multi-site studies because they can handle many types of unmeasured dependence among outcomes. All statistical analyses were conducted using SAS version 9.2 (SAS institute, Cary, NC, USA).

### 2.5. Ethics Statement

Official approval was obtained from the National Health Insurance Corporation in Korea to use the data in the present study (official document no. NHIS-2021-1-526). This study was exempted from the need to obtain approval from the institutional review board (IRB no. EUIRB2020-067) because it used public National Health Insurance claims data. The data were tagged using temporary IDs in order to prevent participants from being identified, and external data leakage was avoided by only performing analyses in a designated research center.

## 3. Results

### 3.1. Patient and Hospital Characteristics

In total, 11,693 patients from 43 tertiary hospitals were analyzed. Table 2 shows the distribution of the hospitals and patients according to institutional characteristics. The most common number of adult ICU beds was less than 50 (32.6%), and the average number of beds was 64 (SD:31). There were 19 (44.2%) hospitals, each with a dedicated resident and a dedicated specialist in the ICU. The majority of the hospitals (*n* = 27; 62.8%) had a grade 2 (0.5 or more and less than 0.63 beds per ICU nurse) adult ICU nurse staffing level, while 10 (23.3%) had grade 1 (less than 0.5 beds per ICU nurse) staffing. A total of 5 hospitals had a grade 3 (0.63 or more and less than 0.77 beds per ICU nurse) nurse staffing level and 1 hospital had a grade 4 (0.77 or more and less than 0.88 beds per ICU nurse) nurse staffing level.

Table 3 presents the characteristics of the study participants. Their average age was 61.2 years, and 59.8% were men. Most patients (67.0%) had a Charlson comorbidity index score of 1–5. Most of the patients were hospitalized through an outpatient visit (i.e., not through the emergency department), and 33.7% of the participants had a history of respiratory disease. In 74.1% of all the participants, the ventilator was applied for less than 1 day.

### 3.2. Univariate Analysis of HAP and In-Hospital Mortality According to Hospital Characteristics

Of the total 11,693 participants, 307 (2.6%) experienced HAP, and 1280 (10.9%) died in the hospital. Table 4 presents the differences in HAP incidence and in-hospital mortality according to hospital characteristics. The incidence of HAP tended to be lower in the hospitals with a large number of ICU beds than in the hospitals with a low number of ICU beds. Likewise, no statistically significant difference was found in the incidence of HAP according to the presence of a dedicated resident or specialist in the ICU. The hospitals with a lower level of nurse staffing had a statistically significantly higher incidence of HAP than the hospitals with a high level of nurse staffing (*p* < 0.001).

The relationship between the in-hospital mortality rate and hospital characteristics was similar to that of HAP incidence. The in-hospital mortality rate in the hospitals with a dedicated specialist in the ICU was 10.2%, which was statistically significantly lower than the rate of 11.4% in the hospitals without a dedicated specialist. The hospitals with a lower level of nurse staffing had statistically significantly higher in-hospital mortality than the hospitals with a high level of nurse staffing.

### 3.3. Univariate Analysis of HAP and In-Hospital Mortality According to Patient Characteristics

Table 5 shows the results of the univariate analysis of HAP and in-hospital mortality according to patient characteristics. The incidence of HAP showed statistically significant positive relationships with age, the number of comorbid diseases, and the ventilator application time. HAP was statistically significantly more likely to develop in men than in women, in patients hospitalized through the emergency room than in those hospitalized through the outpatient department, and in those with a history of respiratory disease. In-hospital mortality showed similar relationships to patient characteristics to those of HAP occurrence, except that the difference in in-hospital mortality according to sex was not statistically significant.

### 3.4. Relationships of Nurse and Physician Staffing with HAP and In-Hospital Mortality after Adjusting for Patient and Hospital Characteristics

The odds ratios (ORs) with 95% confidence intervals (CIs) for HAP and in-hospital mortality after adjusting for covariates are listed in Table 6. Higher nurse staffing levels were associated with lower rates of HAP incidence and in-hospital mortality. Compared to the hospitals with a lower nurse staffing level (grade 3+), the patients admitted to the hospitals with a high nurse staffing level (grade 1) had a statistically significantly lower risk of HAP (OR: 0.27, 95% CI: 0.09–0.86). The presence of a dedicated resident or specialist in the ICU was not statistically significantly associated with the incidence of HAP. Compared to the hospitals with a lower nurse staffing level (grade 3+), the odds of in-hospital mortality were significantly lower for the patients admitted to the hospitals with high nurse staffing levels (grade 1) (OR: 0.41, 95% CI: 0.18–0.95). The availability of a dedicated resident in the ICU did not show a statistically significant association with in-hospital mortality; however, the risk of in-hospital mortality for the patients in the hospitals with a dedicated specialist in the ICU was lower than that in the hospitals with no dedicated specialist (OR: 0.54, 95% CI: 0.38–0.77).

## 4. Discussion

In this study, we evaluated the association of the nurse staffing level and physician staffing with the health outcomes of patients on a ventilator in adult ICUs after undergoing surgery at tertiary hospitals throughout Korea using health insurance data. The proportion of patients who developed HAP during hospitalization was 2.6% and the rate of in-hospital mortality was 10.9%. The nurse staffing levels showed consistent associations with both patient outcomes (HAP and in-hospital mortality) after controlling for patient and hospital characteristics. Compared to the hospitals with lower ICU nurse staffing levels (grade 3+), the hospitals with higher staffing (grade 1) showed statistically significantly lower rates of HAP incidence and in-hospital mortality. This is in accordance with the results of several previous studies showing that higher levels of nurse staffing in the ICU were associated with better patient outcomes [5,9,11]. In a Chinese study involving 2820 secondary and tertiary hospital ICUs, hospitals with a bed-to-nurse ratio of 0.5 or higher had a significantly higher mortality rate than hospitals with a bed-to-nurse ratio of less than 0.5, but there was no significant difference in the incidence of ventilator-associated pneumonia [27]. However, a direct comparison with this study would be limited due to the differences in hospital level and patient composition.

The ICUs of tertiary hospitals are responsible for treating the most severely ill patients. Because inadequate nurse staffing can negatively affect patient safety, in terms of patients’ conditions, complications, and mortality, it will be necessary to enhance the nurse staffing level to grade 1, which corresponds to fewer than 0.5 beds per nurse. According to the report on the status of ICUs in Korea, the mean number of patients during the daytime is 2.7 per working nurse [28]. In developed countries, it is recommended or mandated for each nurse working in the ICU to care for fewer than 2.0 patients [11,20,29]. This means that even in the ICUs of tertiary hospitals with the highest staffing (grade 1) in Korea, the nurses cared for more patients than the minimum standards in some states in the United States. According to the medical law enforcement regulations, the average number of hospitalized patients per day per ICU nurse per day should not exceed 1.2. This is considered to be a very low standard because it corresponds to grade 5 of the ICU nursing staff at a tertiary hospital.

In our study, the presence of a dedicated resident in the ICU did not show a meaningful association with patient outcomes. The presence of a dedicated specialist (intensivist) in the ICU likewise did not show a relationship with HAP incidence, but it did have a statistically significant association with reduced in-hospital mortality. This may imply that the dedicated ICU physician should be an intensivist with the ability to solve high-risk urgent problems arising from patients in the ICU. In a review, ICUs with an intensivist showed a 15% to 60% relative reduction in mortality compared to ICUs without an intensivist [30]. Management fees for dedicated residents and specialists in the ICU in Korea were introduced in 2008 and 2015, respectively. However, the differences in ICU management charges are not nearly sufficient to entice hospital administrators to invest in better patient care by employing more nurses per unit [6]. In 2015, the Korean government mandated the presence of an intensivist in ICUs of tertiary hospitals. However, general hospitals do not have similar regulations.

The reimbursement rates in Korea are estimated to cover 57.9% to 62.2% of the original costs of critical care [6]. For this reason, hospitals tend to avoid investing in human resources in their ICUs [19]. The government’s reimbursement policy should be improved to enable the employment of a sufficient number of nurses and specialists in ICUs [4]. In the financial incentive system for ICUs in Korea, the amount of addition or subtraction of nursing management fees according to the nursing level has recently been slightly increased, but a more substantial increase is requested [28]. Adequate nurse staffing is not an unnecessary expense but an investment that improves outcomes and reduces complications. In Europe, there are large variations in the number of working personnel, qualifications, and continuing education in critical care by country [11]. Medical institutions may have their own training programs; however, Korea does not require specific qualifications or educational programs for nurses in critical care. Reasonable and acceptable standards for critical care should be established. Currently, there are clinical nurse specialists in critical care in Korea, but there is no separate management fee for their care. More clinical nurse specialists in critical care should be nurtured and the cost of reimbursement for their care should be addressed. As in the UK guidelines [31] for ICU service provision, standards and recommendations for ICU personnel, education, and management methods for various situations need to be prepared in Korea.

A strength of this study is that it reports generalizable findings obtained through an analysis of data from 43 tertiary hospitals nationwide, using health insurance data. In particular, the data on deaths were highly accurate as they were linked with the national death statistics. Based on the surgical code, anesthesia code, ICD-10 disease code, age, and other factors recorded in the claims data, the Health Insurance Review and Assessment Service codes the KDRG for all inpatients. Therefore, this method has the advantage of consistency in the classification of patients. Another strength of this study is that it controlled for various patient and hospital factors that could affect patient outcomes.

### Limitations and Suggestions

However, some limitations of this study should be acknowledged. First, the accuracy of the diagnosis of HAP may have been imperfect because this study used claims data. Patients suspected of having HAP were included in the calculation of the HAP incidence rate, which may have led to an overestimation of the HAP incidence. Although we measured the occurrence of HAP in our study, it was not confirmed whether it occurred 2 days after hospitalization, so our definition is not consistent with the definitions used in previous studies. Therefore, it is necessary to be cautious when comparing our findings with other studies. Nonetheless, the identification of patients with suspected HAP is meaningful from the standpoint of hospital infection management.

A second problem is that it was difficult to accurately determine patients’ preoperative condition, although we tried to capture this information using the Charlson comorbidity index and history of respiratory diseases. Third, the time taken for surgery may affect patient outcomes, but there was no available information on this factor; however, the analysis by surgery type addressed this issue to some extent. Fourth, because this study used claims data, there was no information on variables that could affect HAP, such as patients’ state of consciousness, the use of sedative medications, and enteral nutrition; therefore, these factors could not be adjusted. Lastly, the analysis was limited to the grade of nurse staffing and the presence or absence of dedicated residents and specialists, because information was not available on nurses’ critical care experience and the number of patients per personnel per shift or workload. The nursing grade was calculated using only data from the second quarter of 2016 and may have changed during the study period. Further research will be needed using more detailed information on ICU staffing. It is necessary to conduct a study using ICU mortality or ventilator-free days, an outcome indicator that is known to have a greater impact on the ICU workforce [5]. A study on the change in in-hospital mortality or HAP incidence according to the level of nurse staffing observed quarterly is proposed. In addition, it will be necessary to expand the scope of the analysis to include general hospitals and other types of patients. Subsequent studies should also evaluate the economic impact of reducing the length of hospitalization, medical expenses, and drug costs. Because the quality and the quantity of nursing personnel can have a significant impact on patient outcomes, a study on the effectiveness of education or training programs for intensive care unit nurses is also suggested.

## 5. Conclusions

Our findings show that a higher level of nursing staff in the ICU is associated with a lower risk of HAP and in-hospital mortality. The presence of a dedicated specialist in the ICU likewise did not show a relationship with HAP incidence, but it did have a statistically significant association with reduced in-hospital mortality. The presence of a dedicated resident in the ICU did not show a meaningful association with patient outcomes. The relationship between dedicated ICU physicians and patient outcomes seems to require additional research using more precise measurements. The legal standards of nurse staffing and dedicated specialists in the ICU unit should be clarified and strengthened in order to improve the quality of care and patient safety in the ICU.

## Figures and Tables

**Table 1:** Nurse staffing levels and ICU nursing management fee criteria according to the nursing staffing grade of the adult ICU in tertiary hospitals.

ICU Nurse Staffing Grade	Nurse Staffing Level (Nurse-to-Bed Ratio)	Nurse-to-Patient Ratio ^†^	Estimated Number of Patients per Shift Working Nurse ^‡^	ICU Nursing Management Fee Criteria (2015)
1	<0.5:1	<0.47	<2.64	1.45 times the standard
2	<0.63:1	<0.59	<3.02	1.3 times the standard
3	<0.77:1	<0.73	<3.70	1.15 times the standard
4	<0.88:1	<0.83	<4.22	Standard fee
5	≥0.88:1	≥0.83	≥4.22	0.85 times the standard

ICU = intensive care unit. ^†^ Nurse-to-bed ratio converted to nurse-to-patient ratio using an occupancy rate of 94.4%. ^‡^ Number of patients per shift-working nurse = nurse-to-bed ratio × 4.8.

**Table 2:** Hospital and patient distribution by institutional characteristics.

Variable	Category	Hospital (*n* = 43)	Patient (*n* = 11,693)
		*n* (%)	*n* (%)
Hospital ownership	Public	10 (23.3)	3708 (31.7)
	Educational foundation	29 (67.4)	5492 (47.0)
	Medical corporation	4 (9.3)	2493 (21.3)
Location	Seoul	14 (32.6)	6209 (53.1)
	Metropolitan cities	15 (34.9)	2452 (21.0)
	Other regions	14 (32.6)	3032 (25.9)
Number of adult ICU beds	<50	14 (32.6)	1862 (15.9)
	50–69	18 (41.9)	2399 (20.5)
	70–119	8 (18.6)	3975 (34.0)
	≥120	3 (7.0)	3457 (29.6)
Dedicated resident in ICU	No	24 (55.8)	6580 (56.3)
	Yes	19 (44.2)	5113 (43.7)
Dedicated specialist in ICU	No	24 (55.8)	7385 (63.2)
	Yes	19 (44.2)	4309 (36.9)
Nurse staffing level in ICU	Grade 1	10 (23.3)	6896 (59.0)
	Grade 2	27 (62.8)	4052 (34.7)
	Grade 3 and higher	6 (14.0)	745 (6.4)

ICU = intensive care unit.

**Table 3:** Characteristics of the study subjects (*n* = 11,693).

Variable	Category	*n* (%)
Age	20–49	2138 (18.3)
	50–59	2832 (24.2)
	60–69	3074 (26.3)
	70–85	3649 (31.2)
Sex	Male	6990 (59.8)
	Female	4703 (40.2)
Korean Diagnosis-Related Group	Major craniotomy (excluding trauma)	3353 (28.7)
	Other type of craniotomy (excluding trauma)	550 (4.7)
	Craniotomy for trauma	627 (5.4)
	Cardiac valve surgery using cardiac catheterization	588 (5.0)
	Cardiac valve surgery without cardiac catheterization	1305 (11.2)
	Coronary artery bypass graft	1799 (15.4)
	Major vascular repair surgery	480 (4.1)
	Percutaneous angioplasty	1272 (10.9)
	Esophageal surgery	505 (4.3)
	Rectal surgery	98 (0.8)
	Major small and large intestine surgery	644 (5.5)
	Gastric and duodenal surgery	273 (2.3)
	Pancreas and liver surgery	199 (1.7)
Charlson comorbidity index	0	1833 (15.7)
	1–5	7836 (67.0)
	≥6	2024 (17.3)
Admission route	Outpatient visit	9156 (78.3)
	Emergency room	2537 (21.7)
History of respiratory disease	No	7756 (66.3)
	Yes	3937 (33.7)
Ventilator application time	<1 day	8662 (74.1)
	1 day to 3 days	2180 (18.6)
	4 days and more	851 (7.3)

**Table 4:** Hospital-acquired pneumonia and in-hospital mortality according to hospital characteristics (*n* = 11,693).

Variable	Category	Incidence of Pneumonia	X^2^ (*p*)	In-Hospital Mortality	X^2^ (*p*)
		*n* (%)		*n* (%)	
Hospital ownership	Public	54 (1.5)	77.28	227 (6.1)	400.47
	Educational foundation	220 (4.0)	(<0.001)	937 (17.1)	(<0.001)
	Medical corporation	33 (1.3)		116 (4.7)	
Location	Seoul	66 (1.1)	193.59	391 (6.3)	345.09
	Metropolitan cities	156 (6.4)	(<0.001)	480 (19.6)	(<0.001)
	Other regions	85 (2.8)		409 (13.5)	
Number of adult ICU beds	<50	79 (4.2)	249.03	416 (22.3)	810.38
	50–69	157 (6.5)	(<0.001)	499 (20.8)	(<0.001)
	70–119	53 (1.3)		286 (7.2)	
	≥120	18 (0.5)		79 (2.3)	
Dedicated resident in ICU	No	171 (2.6)	0.04	738 (11.2)	1.12
	Yes	136 (2.7)	(0.838)	542 (10.6)	(0.290)
Dedicated specialist in ICU	No	202 (2.7)	0.95	842 (11.4)	4.28
	Yes	105 (2.4)	(0.330)	438 (10.2)	(0.039)
Nurse staffing level in ICU	Grade 1	41 (0.6)	278.31	263 (3.8)	952.81
	Grade 2	214 (5.3)	(<0.001)	791 (19.5)	(<0.001)
	Grade 3 and higher	52 (7.0)		226 (30.3)	

ICU = intensive care unit.

**Table 5:** Hospital-acquired pneumonia and in-hospital mortality according to patient characteristics (*n* = 11,693).

Variable	Category	Incidence of Pneumonia	X^2^ (*p*)	In-Hospital Mortality	X^2^ (*p*)
		*n* (%)		*n* (%)	
Age	20–49	31 (1.5)	34.9	175 (8.2)	139.91
	50–59	65 (2.3)	(<0.001)	253 (8.9)	(<0.001)
	60–69	71 (2.3)		268 (8.7)	
	70–85	140 (3.8)		584 (16.0)	
Sex	Male	201 (2.9)	4.25	769 (11.0)	0.05 (0.817)
	Female	106 (2.2)	(0.039)	511 (10.9)	
Korean Diagnosis-Related Group	Major craniotomy (excluding trauma)	69 (2.1)	364.56	298 (8.9)	1604.28
	Other type of craniotomy (excluding trauma)	32 (5.8)	(<0.001)	232 (42.2)	(<0.001)
	Craniotomy for trauma	38 (6.1)		210 (33.5)	
	Cardiac valve surgery using cardiac catheterization	1 (0.2)		2 (0.3)	
	Cardiac valve surgery without cardiac catheterization	5 (0.4)		5 (0.4)	
	Coronary artery bypass graft	4 (0.2)		5 (0.3)	
	Major vascular repair surgery	1 (0.2)		29 (6.0)	
	Percutaneous angioplasty	114 (9.0)		273 (21.5)	
	Esophageal surgery	4 (0.8)		5 (1.0)	
	Rectal surgery	2 (2.0)		12 (12.2)	
	Major small and large intestine surgery	22 (3.4)		133 (20.7)	
	Gastric and duodenal surgery	14 (5.1)		49 (18.0)	
	Pancreas and liver surgery	1 (0.5)		27 (13.6)	
Charlson comorbidity index	0	28 (1.5)	16.27	190 (10.4)	34.97
	1–5	206 (2.6)	(<0.001)	793 (10.1)	(<0.001)
	≥6	73 (3.6)		297 (14.7)	
Admission route	Outpatient visit	181 (2.0)	69.45	744 (8.1)	344.47
	Emergency room	126 (5.0)	(<0.001)	536 (21.1)	(<0.001)
History of respiratory disease	No	115 (1.5)	117.67	794 (10.2)	11.89
	Yes	192 (4.9)	(<0.001)	486 (12.3)	(0.001)
Ventilator application time	<1 day	168 (1.9)	118.92	579 (6.7)	1349.13
	1 day to 3 days	70 (3.2)	(<0.001)	296 (13.6)	(<0.001)
	4 days and more	69 (8.1)		405 (47.6)	

**Table 6:** Logistic regression analysis of hospital-acquired pneumonia and in-hospital mortality.

			Incidence of Pneumonia		In-Hospital Mortality	
	Variable	Category	OR	(95% CI)	OR	(95% CI)
Patient factors	Age		1.01	(1.00–1.03)	1.02	(1.01–1.03)
	Sex	Male	1.30	(1.04–1.62)	1.03	(0.88–1.21)
		Female	1		1	
	Charlson comorbidity index	0	0.58	(0.33–1.01)	0.84	(0.62–1.14)
		1–5	0.84	(0.62–1.13)	0.82	(0.66–1.01)
		≥6	1		1	
	History of respiratory disease	Yes	3.86	(2.85–5.23)	1.06	(0.87–1.28)
		No	1			
	Admission route	Outpatient visit	0.53	(0.40–0.71)	1.32	(1.07–1.63)
		Emergency room	1		1	
	Ventilator application days	1–3	1.07	(1.04–1.11)	1.73	(1.29–2.31)
		≥4	1.46	(1.03–2.08)	6.13	(4.21–8.92)
		<1	1		1	
Hospital factors	Hospital ownership	Public	0.61	(0.39–0.95)	0.60	(0.30–1.20)
		Educational foundation	0.96	(0.60–1.55)	0.96	(0.56–1.64)
		Medical corporation	1		1	
	Location	Seoul	0.78	(0.34–1.77)	0.97	(0.60–1.58)
		Metropolitan cities	1.61	(0.71–3.66)	1.16	(0.80–1.69)
		Other regions	1		1	
	Number of adult ICU beds		1	(0.99–1.01)	0.99	(0.99–1.00)
	Dedicated resident in ICU	Yes	0.93	(0.50–1.72)	0.82	(0.58–1.17)
		No	1		1	
	Dedicated specialist in ICU	Yes	0.92	(0.53–1.59)	0.54	(0.38–0.77)
		No	1		1	
	Nurse staffing level in ICU	Grade 1	0.27	(0.09–0.86)	0.41	(0.18–0.95)
		Grade 2	0.90	(0.36–2.23)	0.79	(0.51–1.22)
		Grade 3 and higher	1		1	

Note: Korean Diagnosis-Related Group categories were adjusted. ICU = intensive care unit.

## Data Availability

Research data are not shared. Data used in this study can only be analyzed by researchers approved by the National Health Insurance Service at a designated research center.
